# Identification and functional analysis of *SWEET* gene family in *Averrhoa carambola* L. fruits during ripening

**DOI:** 10.7717/peerj.11404

**Published:** 2021-05-31

**Authors:** Qihua Lin, Qiuzhen Zhong, Zehuang Zhang

**Affiliations:** Fruit Research Institute, Fujian Academy of Agricultural Sciences, Fuzhou, China

**Keywords:** *Averrhoa Carambola*, *AcSWEETs*, Expression patterns, Sugar transport

## Abstract

Sugar Will Eventually be Exported Transporters (*SWEETs*), a type of sugar efflux transporters, have been extensively researched upon due to their role in phloem loading for distant sugar transport, fruit development, and stress regulation, etc. Several plant species are known to possess the *SWEET* genes; however, little is known about their presence in *Averrhoa Carambola* L. (Oxalidaceae), an evergreen fruit crop (star fruit) in tropical and subtropical regions of Southeast Asia. In this study, we established an *Averrhoa Carambola* L. unigenes library from fruits of ‘XianMiyangtao’ (XM) by RNA sequencing (RNA-seq). A total of 99,319 unigenes, each longer than 200 bp with a total length was 72.00 Mb, were identified. A total of 51,642 unigenes (52.00%) were annotated. Additionally, 10 *AcSWEET* genes from the *Averrhoa Carambola* L. unigenes library were identified and classified, followed by a comprehensive analysis of their structures and conserved motif compositions, and evolutionary relationships. Moreover, the expression patterns of *AcSWEETs* in ‘XM’ cultivars during fruit ripening were confirmed using quantitative real-time PCR (qRT-PCR), combined with the soluble sugar and titratable acids content during ripening, showed that *AcSWEET2a/2b* and *AcSWEET16b* might participate in sugar transport during fruit ripening. This work presents a general profile of the *AcSWEET* gene family in *Averrhoa Carambola* L., which can be used to perform further studies on elucidating the functional roles of *AcSWEET* genes.

## Introduction

In higher plants, the end-products of photosynthesis include glucose, fructose, and sucrose. These sugars provide a carbon skeleton for large molecules, such as proteins and nucleic acids, are used as osmolytes and signaling molecules, and are temporarily stored in plant vacuoles and transported to different organs as nutrients for cellular growth and development (Ayre, 2011; Wind, Smeekens & Hanson, 2010; Braun, 2012). Currently, sucrose transporters (*SUTs*), monosaccharide transporters (*MSTs*), and Sugar Will Eventually be Exported Transporters (*SWEETs*) are commonly used for sugar translocation (Chen et al., 2015a; Eom et al., 2015).

The *SWEET* gene family was first discovered in Arabidopsis (Chen et al., 2010). They are known to play diverse physiological roles, such as phloem loading for distant sugar transport (Braun, 2012; Zhang et al., 2019), seed filling (Sosso et al., 2015), nectar secretion (Lin et al., 2014), fruit development (Chong et al., 2014; Zheng et al., 2014), pollen nutrition (Sun et al., 2013), plant-pathogen interaction (Chen, 2014; Zhou et al., 2015), and stress regulation (Chandran, 2015; Yue et al., 2015). They are characterized by seven transmembrane helices, composed of two 3-transmembrane-helix MtN3 motifs, which are tandemly connected by a link transmembrane helix (Xuan et al., 2013). They mainly transport hexoses or sucrose across the plasma membranes, thus acting as sugar efflux transporters (Chen et al., 2010; Chen et al., 2012). Since appropriate carbohydrate distribution is vital for crop yield and quality; thus, it is necessary to evaluate the functional role of *SWEET* genes in plants, especially in fruit-bearing trees.

In plants, *SWEET* genes have been classified into the following four subclades depending on their functional properties: clade I and II proteins transport hexose, clade III proteins mainly transport sucrose and minor quantities of fructose, clade IV proteins transport both monosaccharides and disaccharides (Chen et al., 2010; Chong et al., 2014; Wei et al., 2014; Eom et al., 2015). The *SWEET* genes belonging to a specific clade may perform similar functions, although they might be different in plants. Previous studies have identified the *SWEET* sugar transporters in several plants, including Arabidopsis (Chen et al., 2010), rice (*Yuanet al., 2013*), and several fruit-bearing trees, such as orange (Zheng et al., 2014), grape (Chong et al., 2014), apple (Wei et al., 2014), pear (Li et al., 2017), loquat (Wu et al., 2017), litchi (Xie et al., 2019), and pineapple (Guo et al., 2018).

*Averrhoa Carambola* L. (family: Oxalidaceae) is an evergreen plant native to tropical and subtropical regions of Southeast Asia and has been grown in China for more than 2000 years. Its ripe fruit is sweet, juicy, and slightly sour, with a pleasant aroma, and has traditional medical efficacy (Hu et al., 2005). The composition of sugars and organic acids in the fruit of Carambola shows significant variation depending on cultivars and the fruit development stages, but the mechanism of sugar transport in Carambola remains to be elucidated.

Here, we developed the Carambola unigenes library from ‘XianMiyangtao’ (‘XM’) fruits, which are a sweet carambola variety. The fruit has high sugar content, good flavor, low acidity and can be eaten fresh. It is one of the main *carambola* varieties currently grown in China. We analyzed protein structure and function, as well as phylogenetic development of ten *AcSWEET* genes that were identified in *A. Carambola*. Additionally, we studied the expression patterns of *AcSWEET* genes during fruit ripening. Based on the results, we constructed a general profile for the *AcSWEET* family, which helped in understanding the complex mechanism of sugar translocation in this fruit-bearing tree.

## Material and Methods

### Plant materials

We collected all fruit samples from the Carambola ‘XM’ trees, growing in Yunxiao county of the Fujian province of China (latitude: 24.0168°; longitude: 117.2753°). The fruits samples were classified into three categories, based on the ripening stage at harvest: young fruits (XM1, 40 d), developing fruits (XM2, 60 d), and ripe fruits (XM3, 80 d) after flowering.

The samples were selected based on their uniform feature with no damage. For each category, we harvested 30 fruits, which were used for the determination of sugar and acid content, RNA-seq, and qRT-PCR. They were randomly assigned to three groups containing ten fruits each. The pulp of these samples was collected, cleaned, cut, flash-frozen in liquid nitrogen, and stored at −80 °C.

### Determination of soluble sugars content and titratable acidity (TA)

The sulfuric acid-anthrone colorimetric method was used for determining the glucose, fructose and sucrose content, following the method of Hu et al. (2005). Titratable acidity (TA) was measured using the NaOH solution, following the method of Zhu et al. (2013). Each experiment was performed in replicates, and data were represented as mean ± SD.

### RNA extraction, sequencing and bioinformatic analysis

Total RNA was extracted following the method of *Shan et al. (2008)*, followed by treatment with DNase (Takara, China). Next, we performed agarose gel electrophoresis (1%) was performed to assess RNA degradation and contamination. A NanoPhotometer^®^ spectrophotometer was used to analyze the purity of the extracted RNA (IMPLEN, USA). Next, a Qubit^®^ RNA Assay Kit in a Qubit^®^ 2.0 Fluorometer was used to determine the RNA concentration (Life Technologies, Carlsbad, CA, USA). Finally, the RNA Nano 6000 Assay Kit using the Bioanalyzer 2100 system was used to assess the integrity of the RNA sample (Agilent Technologies, USA).

An Illumina Hiseq 4000 platform was used to generate 150 bp/200 bp paired-end reads (Biomarker Technologies Co., China). Next, using Trinity, this data was processed and assembled, resulting in the formation of the Carambola unigenes library. BLAST database (v2.2.26) was used to compare the unigenes sequences with Nr, a manually annotated and reviewed protein sequence database (Swiss-Prot), Clusters of Orthologous Groups (COG), gene ontology (GO), EuKaryotic Ortholog Groups (KOG), protein family (Pfam), evolutionary genealogy of genes: non-supervised orthologous groups of proteins (eggNOG), and Kyoto Encyclopedia of Genes and Genomes (KEGG) using *E* value ≤ E^−5^. The gene expression was quantified as Fragments per kilobase per million fragments (FPKM). Transcriptome data has been submitted to National Center for Biotechnology Information (NCBI) database (SRA accession: PRJNA647672), and this Transcriptome Shotgun Assembly project has been deposited at DDBJ/EMBL/GenBank under the accession GJAU01000000. The version described in this paper is the first version, GJAU00000000.1.

### Identification of *SWEET* gene family members

We used the Hidden Markov Model (HMM) analysis and a Simple Modular Architecture Research Tool (SMART). The HMM profile was downloaded from Pfam protein family database (http://pfam.xfam.org/) to obtain the Pfam number of protein sequence. We used the HMM search command in the HMMER package, with an e-value ≤1e^−3^ to identify the protein domain. The results of the HMMER sequence alignment were screened to remove protein sequences that were 45% shorter than the length of the HMM model domain, while retaining the longest protein sequence in the variable shear. All nonredundant protein sequences were retrieved and further analyzed with SMART (a Simple Modular Architecture Research Tool; http://smart.embl-heidelberg.de/) to examine the results. These genes were confirmed as family members.

### Protein domain prediction, similarity analysis and phylogenetic analysis

HMMER-profile hidden Markov models were used for biological sequence analysis (http://hmmer.org/) and to predict protein domains of sweet genes. The results of the alignment were output by Clustal 2.1. Transmembrane (TM) structures were detected by using TMHMM (http://www.cbs.dtu.dk/services/TMHMM/) and ConPred II (Arai et al., 2004). Protparam tool was used to predict the molecular weight and isoelectric point and ClustalW 2 was used to construct the phylogenetic tree of *SWEET* gene family.

### Quantitative real-time PCR analysis (qRT-PCR)

The expression profile for each member of the *AcSWEET* gene family was determined through qRT-PCR using the 2^−ΔΔ*Ct*^ method with glyceraldehyde-3-phosphate dehydrogenase *(AcGAPDH)* as the reference gene. We used the Data Processing System v3.01 to calculate the least significant differences (*a* = 0.05) for the mean separations. All experiments were performed in triplicates. File S1 provides a list of the gene primer sequences for qRT-PCR.

### Statistical analysis

All statistical analyses were performed using SPSS Statistics software 19.0 (SPSS Inc., USA) for Windows, and the values were represented as mean ± SE.

## Results

### Sequencing production statistics and de novo assembly

The total RNA of XM1, XM2, and XM3 fruits (*n* = 3) was used to construct nine cDNA libraries. Next, we performed sequencing quality control on the raw reads that were obtained from nine sequencing libraries, which resulted in 78.53 Gb of clean data. The results showed that the rate of accordance with Q30 of each sample was greater than 94%, and the rate of GC was in the range of 44.8–46%. Thus the sequencing data obtained in this study were of good quality.

Next, we performed de novo assembly using Trinity and obtained 99,319 unigenes using the Trinity reads. Based on the length distribution of all unigenes, we found that the minimum length of unigenes was 200 bp. Maximum unigenes had 200 to 300 bp (38,786; 39.05%), followed by 300 to 500 bp (26,709; 26.89%), 500 to 1000 bp (16,902; 17.02%), 1000 to 2000 bp (8,470; 8.53%), and >2000 bp (8,452; 8.51%) (File S2). All the 99,319 unigenes were 72.0 Mb in length, with an average length of 725.28 bp and N50 was 1374 bp, further indicating that the splicing result was accurate (Table 1).

**Table 1:** Results of sequencing and de novo assembly.

Sequences	XM1	XM2	XM3
Post-trimming			
Number of clean reads	25,493,659	28,145,875	34,365,857
GC content (%)	45.59	45.63	45.01
Q30 percentage (%)	95.02	95.22	95.66
Post-assembly			
Number of transcripts	189,432		
Mean length of transcripts	1,426.80		
N50 of transcripts (bp)	2,576		
Number of unigenes	99,319		
Mean length of unigenes (bp)	725.28		
N50 of unigenes (bp)	1,374		

### Functional annotation

**Figure 1:**
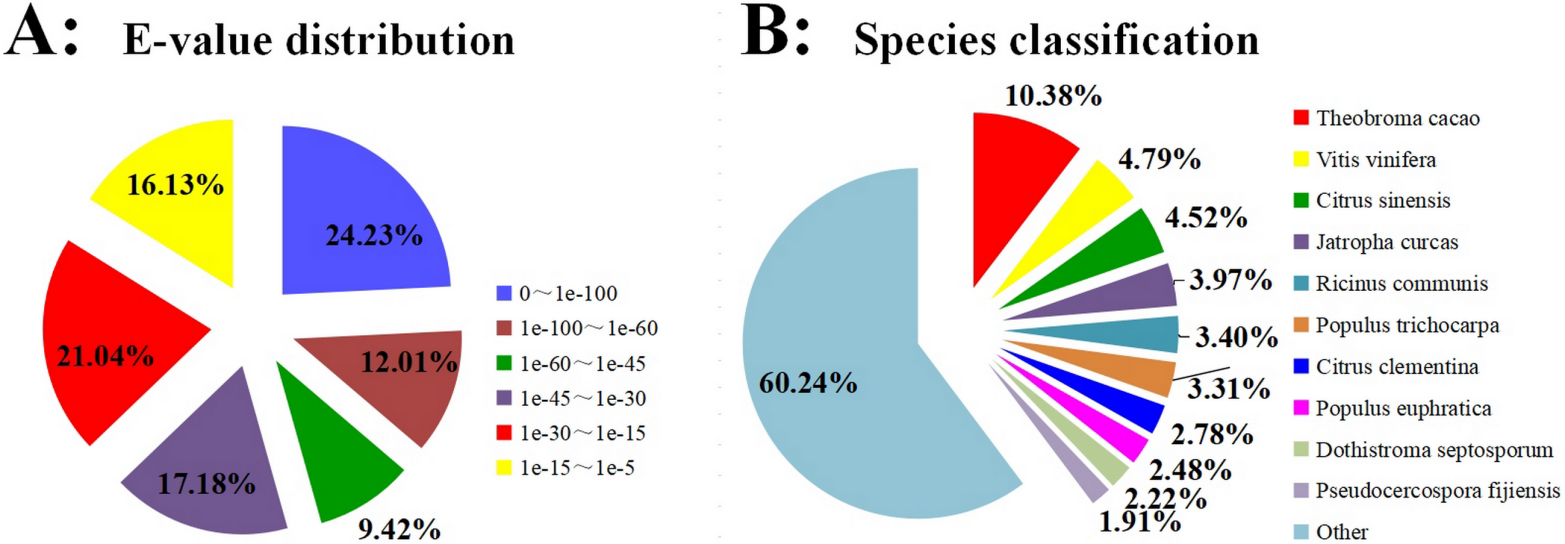
Homology searches of Carambola unigenes. (A) For each unigenes, the distribution of E-value of the top BLASTx hits against the Nr database. (B) Percentages of unigenes in sequence alignment from the top ten species using BLASTx against the Nr database.

BLASTX was used to annotate all the 99,319 unigenes sequences against the Nr, GO, Swiss-Prot, KOG, COG, Pfam, eggNOG, and KEGG for comprehensive analysis. The annotation of 51,642 unigenes (52.00%) was performed with a significance threshold of the BLAST parameter *E*-value less than 1e^−5^ and HMMER parameter *E*-value less than 1e^−10^ (Fig. 1A). The homology sequence alignment analyses showed that 41,386 (41.7%), 48,193 (48.5%), 32,475 (32.7%), 31,280 (31.5%), 24,794 (25. 0%), 23,556(23.7%), 16,534 (16.6%), and 15,896 (16.0%) unigenes had homologous sequences in the Nr, eggNOG, Pfam, KOG, GO, Swiss-Prot, KEGG, and COG, respectively (Table 2). The results also revealed that approximately 11,135 (9.30%) unigenes showed sequence alignment with all databases. Due to the shortage of relevant genetic data, 48.00% of unigenes could not be annotated. Based on the results of sequence alignment, the following species showed sequence similarity with Carambola: *Theobroma cacao* (10.38%), *Vitis vinifera* (4.79%), *Citrus sinensis* (4.52%), *Jatropha curcas* (3.97%), *Ricinus communis* (3.40%), *Populus trichocarpa* (3.31%), *Citrus clementina* (2.78%), *Populus euphratica* (2.48%), *R. Dothistroma septosporum* (2.22%), and *Pseudocercospora fijiensis* (1.91%) (Fig. 1B).

**Table 2:** Annotations of the assembled Carambola unigenes using public database sequences.

Anno_Database	Annotated_Number	300< = length<1000	length> = 1000
NR_Annotation	41386	14191	14421
eggNOG_Annotation	48193	18378	14605
Pfam_Annotation	32475	12071	12574
KOG_Annotation	31280	12402	9629
GO_Annotation	24794	7735	9213
Swissprot_Annotation	23556	7993	10357
KEGG_Annotation	16534	6125	5803
COG_Annotation	15896	5170	5899
All_Annotated	51642	19546	14993

### GO classification

The gene ontology (GO) database is a universal method for the functional classification of genes. It is used to comprehensively understand the functional characteristics of biologically expressed genes in different organisms. Here, the GO database was used to categorize standardized gene functions of the expressed Carambola unigenes. The results of the Blast2GO and WEGO software analysis revealed that 24,794 unigenes out of 41,386, which had been previously annotated to the Nr database, were classified into three main GO categories (molecular function, biological process, and cellular component) and 55 subcategories (File S3). The major subcategories among the molecular function were ‘Catalytic activity’, which was probably related to the continuous cell proliferation during fruit development and the vigorous metabolic activity in fruit organs.

### Differentially expressed genes

**Figure 2:**
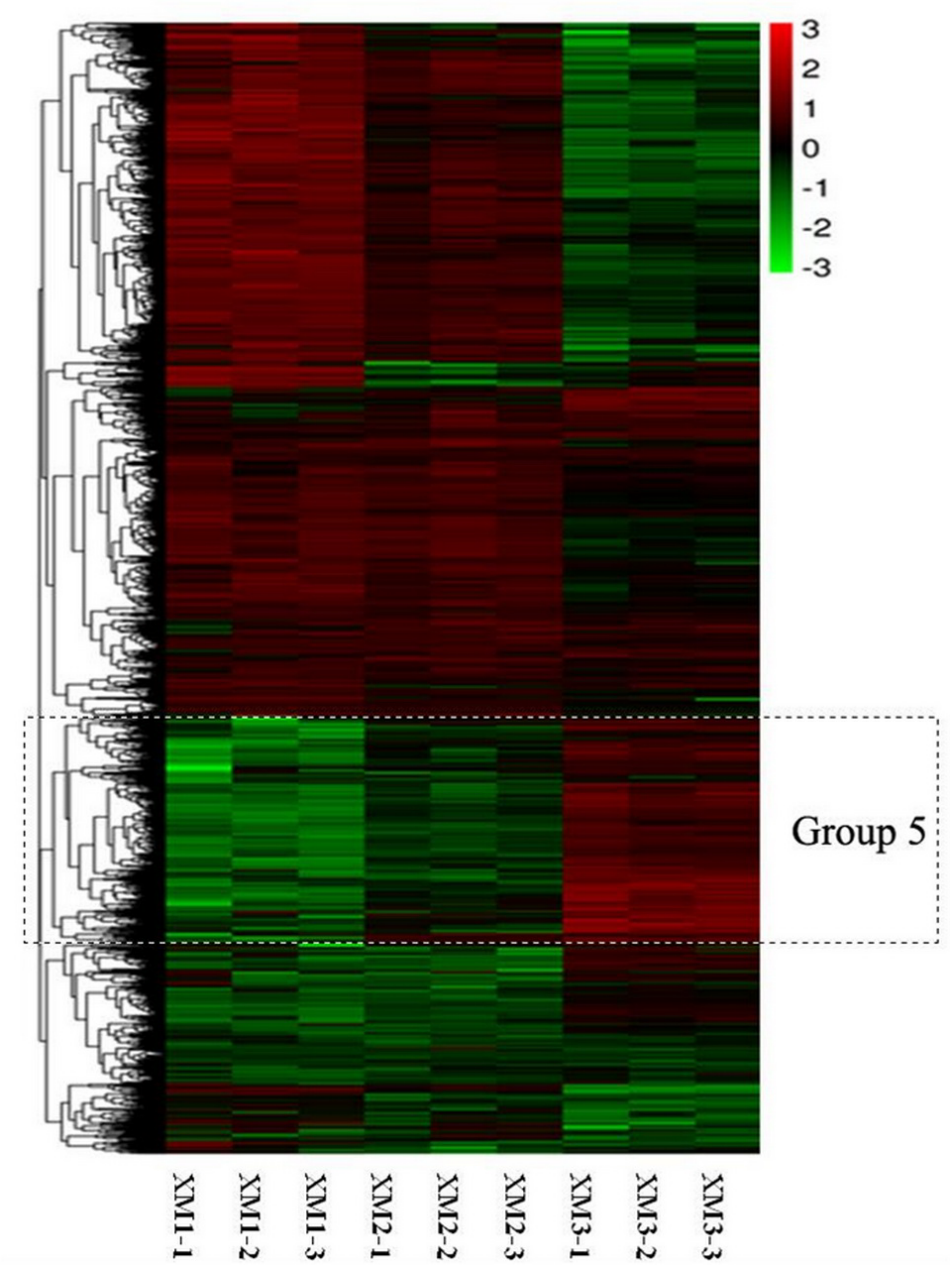
Heatmap analysis of DEGs at three distinct ripening stages of Carambola. Each stage consists of three repetitions.

The RNA-Seq analysis revealed that the numbers and expression profiles of DEGs differed at three stages. These DEGs were clustered into seven distinct expression patterns, among which group 5 were all upregulated from the stage 1 to stage 3 (Fig. 2). Group 5 contained several genes related to sugar metabolism, including sucrose metabolic process, sucrose-phosphate synthase activity. Thus group 5 were closely related to fruit ripening.

### Identification of *SWEET* genes in Carambola

The putative Carambola *SWEETs* were identified by protein Blast of the PFAM motif PF03083 against Carambola unigenes library and from the annotation of the Carambola unigenes library. As shown in File S4, we obtained 10 *SWEET* genes, each containing MtN3/saliva domains (PFAM motif PF03083) in *A. Carambola*. Simultaneously, from the Nr annotation data of unigenes, we obtained 12 *SWEET* genes, of which 10 *SWEET* genes were consistent with the above identification. The other two *SWEET* genes were unigene c94236.graph_c0 and c89229.graph_c0 and their annotation references were *SWEET5* and *SWEET13*, respectively. Due to extremely low expression of these two *SWEET* genes in Carambola fruit along with the fact that they were derived only from the NR annotation information, they were not identified as *SWEET* genes in this study.

The *SWEET* genes in Carambola fruit, hereafter referred to as *AcSWEETs*, were labeled as *AcSWEET1a*, *AcSWEET1b*, *AcSWEET2a*, *AcSWEET2b*, *AcSWEET3*, *AcSWEET11*, *AcSWEET15*, *AcSWEET16a*, *AcSWEET16b*, and *AcSWEET17*, based on their percentage of similarity to Arabidopsis, Oryza, and Vitis *SWEET* genes. MAFFT analysis showed that the sequences of these ten SWEET proteins were different from each other (File S5). Among these SWEET proteins, AcSWEET15 was the longest, i.e., contained 305 amino acids, while AcSWEET2a contained only 183 amino acids (File S4).

### Protein domain prediction of the SWEET proteins in Carambola fruit

**Figure 3:**
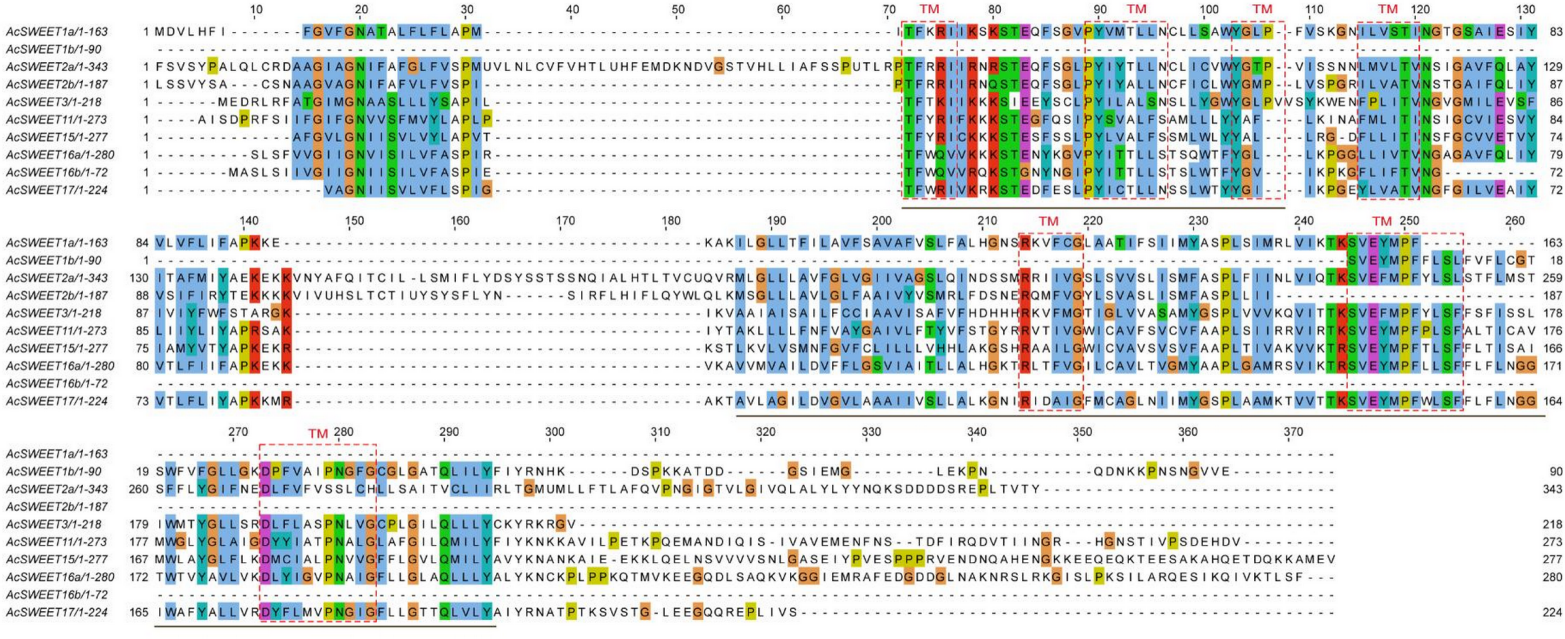
Homology comparison of predicted amino acid sequence of AcSWEETs. The putative transmembrane regions are boxed and the conservative MtN3/slv domain are underlined.

Transmembrane (TM) sequences were found to be partially conserved, based on the results of multiple sequence alignment of the ten AcSWEET protein sequences. The results of protein annotation revealed that all ten proteins contained the MtN3/slv motif, which was conservative in plant SWEETs. Also, AcSWEET1a, AcSWEET2a, AcSWEET2b, AcSWEET3, AcSWEET11, AcSWEET15, AcSWEET16a, and AcSWEET17 contained two MtN3/slv conserved domains, while AcSWEET1b, and AcSWEET16b had only one MtN3/slv domain (Fig. 3), as the AcSWEET sequence were all from Carambola unigenes, not full-length genes, consistent with the results in loquat.

Using TMHMM and ConPred II, we found that all the Carambola SWEET proteins contained TM structures. Seven TMs were detected in AcSWEET2a, AcSWEET3, AcSWEET11, AcSWEET15, AcSWEET16a, and AcSWEET17. However, only six, two, five, and four TMs were identified in AcSWEET1a, AcSWEET1b, AcSWEET2b, and AcSWEET16b, respectively (Fig. 3).

### Phylogenetic analysis of putative SWEET proteins in Carambola

**Figure 4:**
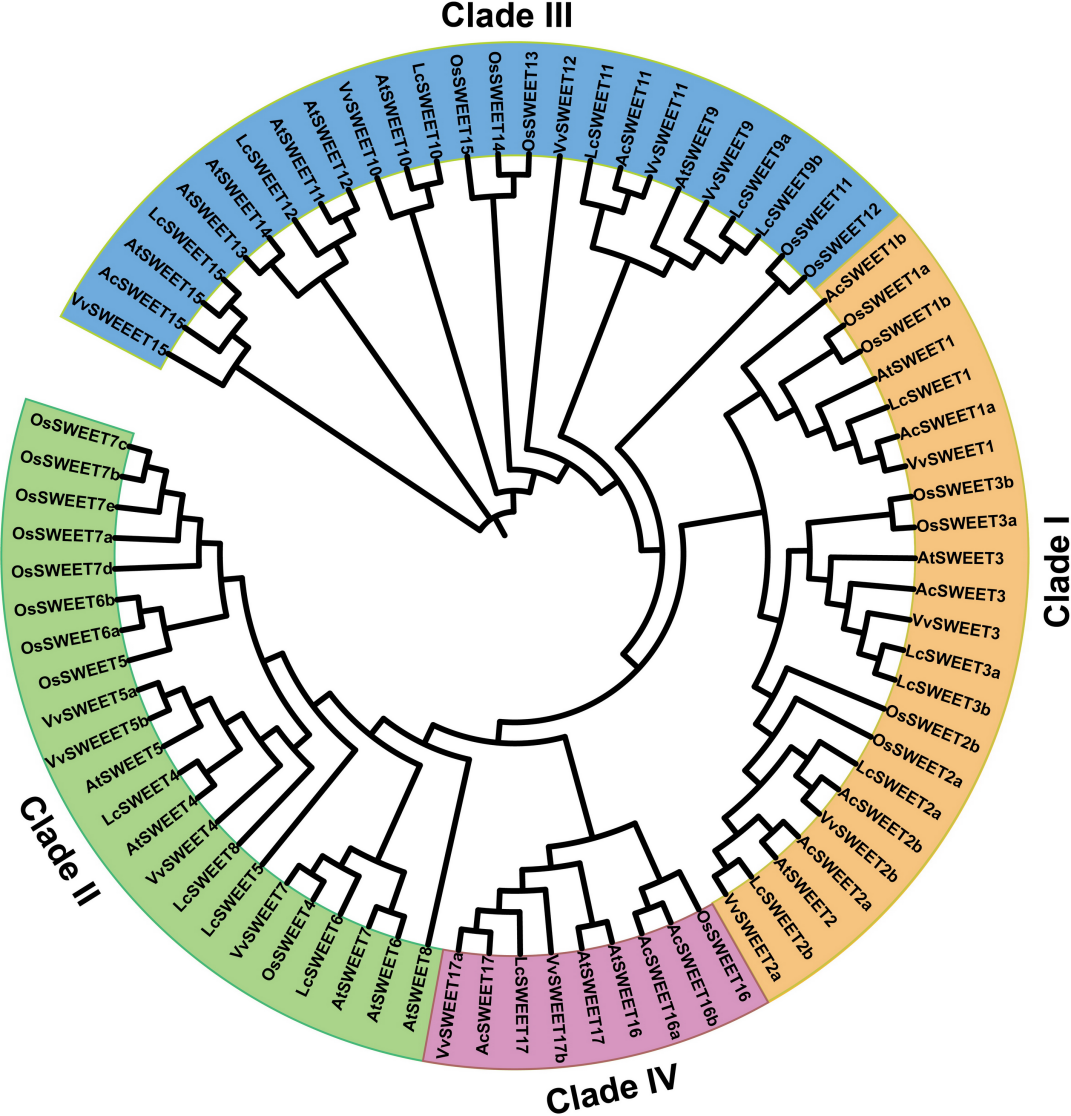
Phylogenetic tree of putative SWEET proteins from plants. File S4 provides the sequences for alignment.

A neighbor-joining phylogenetic tree was constructed using MEGA 7 software to explore the evolutionary relationships between SWEET proteins from other plant species and AcSWEET identified in this study. We obtained a total of 79 amino acid sequences of SWEET proteins, including 10 AcSWEET sequences, 17 AtSWEET sequences, 21 OsSWEET sequences, 15 VvSWEET sequences, and 16 LcSWEET sequences (File S4). Based on the results, the SWEET proteins were classified into four different clades (Fig. 4), with clade I containing AcSWEET1a/1b/2a/2b/3, clade III contained AcSWEET11/15, clade IV contained AcSWEET16a/16b/17, but none of the AcSWEET proteins was found in clade II. AcSWEET1a, AcSWEET2b, and AcSWEET17 showed high sequence similarity with VvSWEET1, VvSWEET2b, and VvSWEET17a, respectively. AcSWEET2a and AcSWEET16b had high sequence similarity with AtSWEET2 and OsSWEET16, respectively.

### Differential expression of the *SWEET* genes in the fruit of Carambola

We used RNA-Seq and qRT-PCR to study the expression of the ten *SWEET* genes in Carambola fruits at three distinct ripening stages: young fruits (XM1, 40 d), developing fruits (XM2, 60 d), and ripe fruits (XM3, 80 d) post-flowering. We found that ten *AcSWEET* genes were expressed differently in three different developmental stages, and *AcSWEET1a/1b/2a/2b/16b/17* had higher expression during fruit development compared with *AcSWEET3/11/15/16a*, which were expressed only at certain stages. Among the six highly expressed *AcSWEET* genes, the expression of *AcSWEET16b* at three different stages of fruit development was much higher than the other five *AcSWEET* genes, indicating that *AcSWEET16b* played a greater role in the entire fruit development process. The expression of *AcSWEET2a/2b* decreased during fruit development, indicating that it might play a role mainly during the early and middle stages of fruit ripening (Table 3).

**Figure 5:**
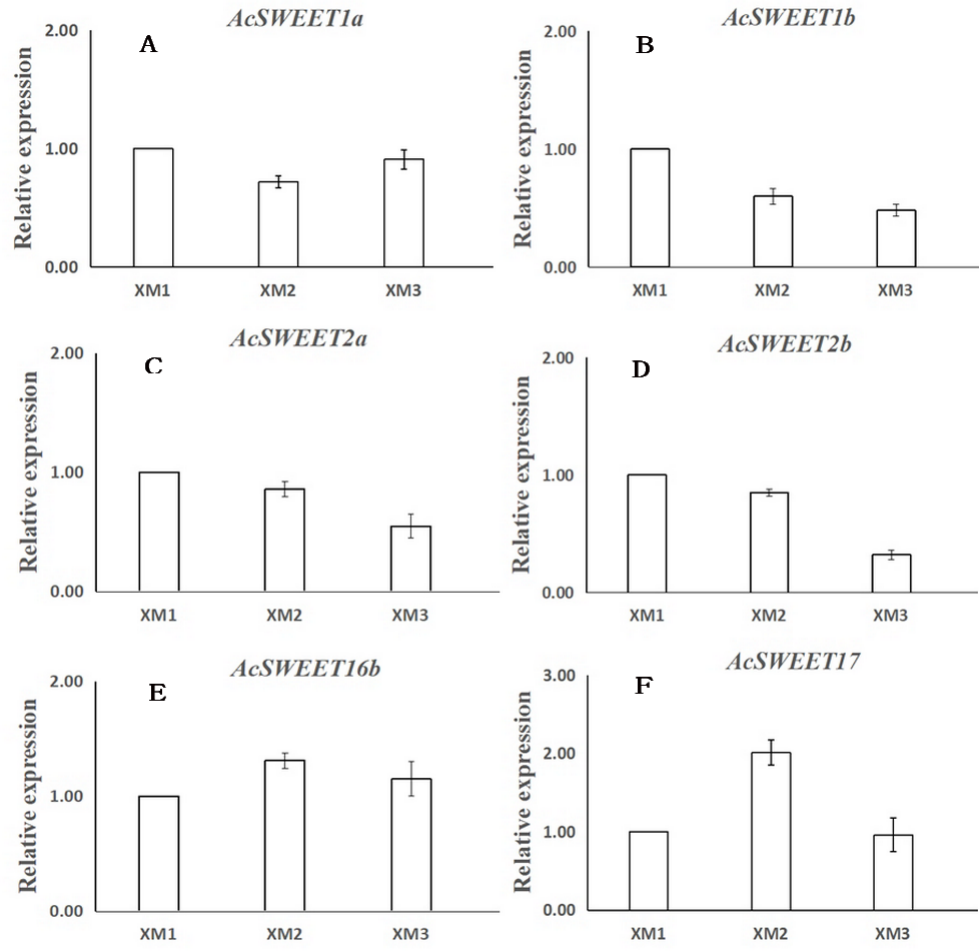
Expression analysis of six *AcSWEET* genes using qRT-PCR. (A–F) respectively represent *AcSWEET1a/1b/2a/2b/16b/17*. The *y*-axis represents the relative expression and the *x*-axis depicts the three different samples (XM1, XM2, and XM3). The vertical bars indicate the standard error of three replicates.

Next, we verified the expression of 6 *AcSWEETs* (*AcSWEET1a/1b/2a/2b/16b/17*) in Carambola using qRT-PCR to validate the accuracy of the RNA-Seq results, which was consistent with our results (Fig. 5 and File S6).

### Soluble sugars content and organic acids in ‘XM’ at distinct ripening stages

**Figure 6:**
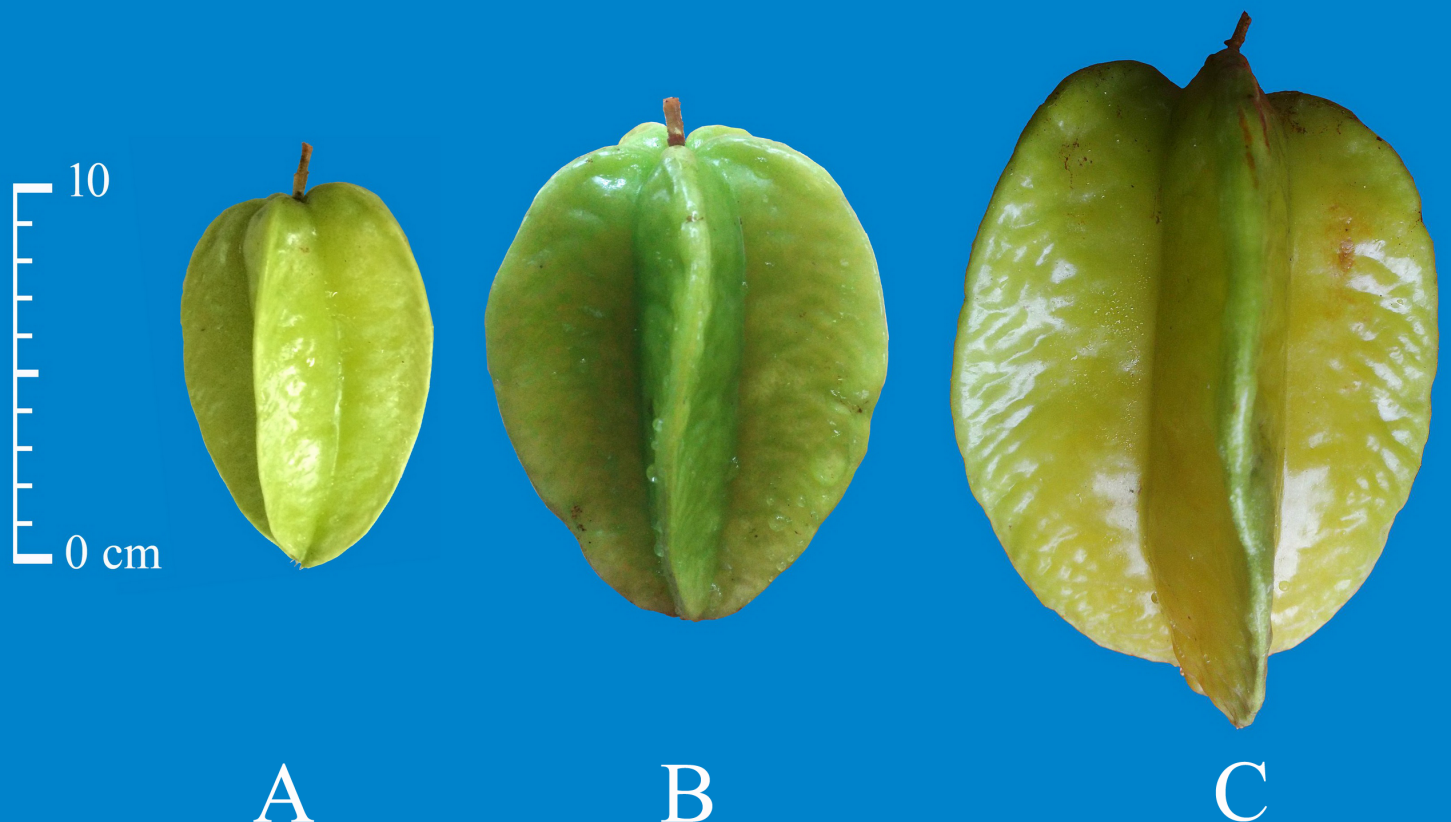
Visual appearance at three distinct ripening stages in the ‘XM’. (A) Young fruits of ‘XM’ at 40 days after flowering (DAF), (B) fruits of ‘XM’ at 60 DAF, (C) ripe fruits of ‘XM’ at 80 DAF.

**Figure 7:**
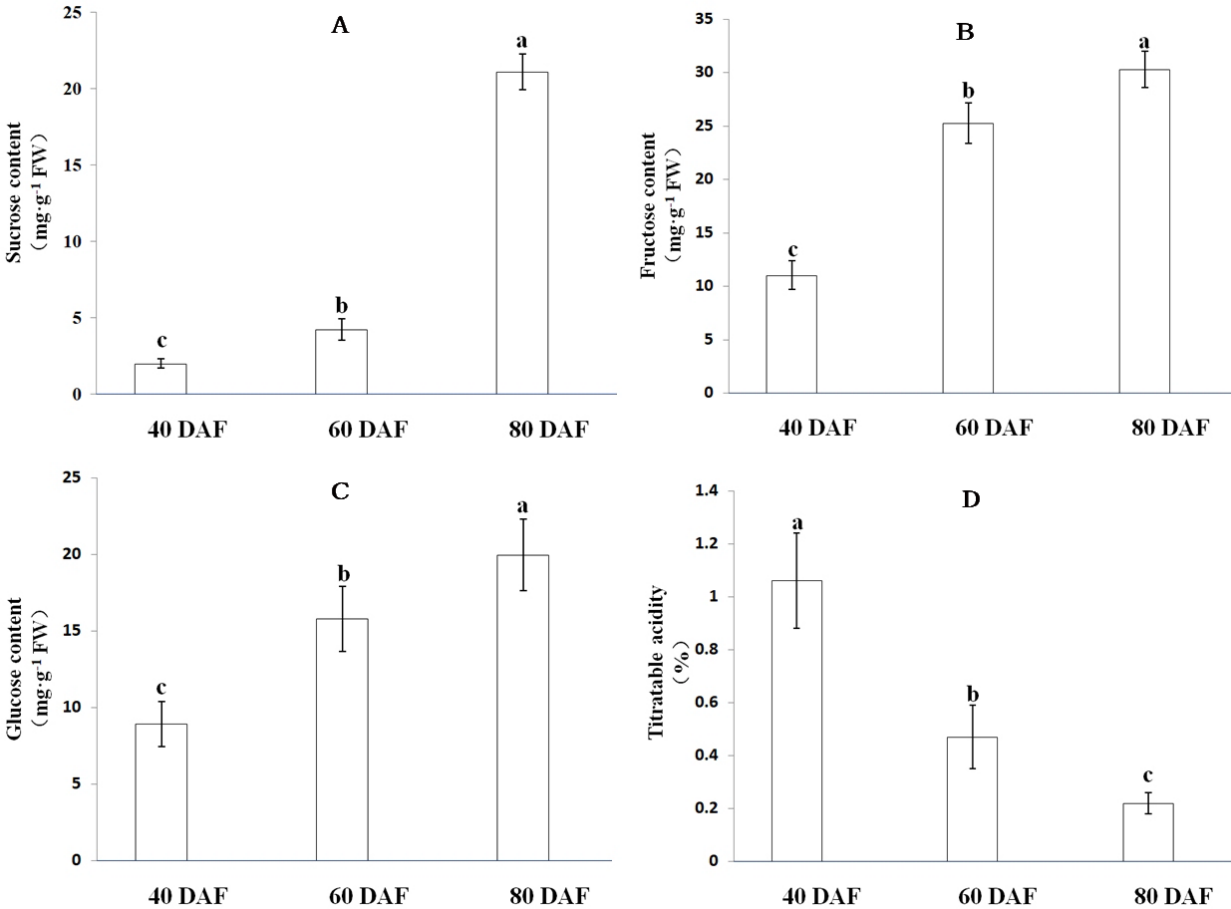
Soluble sugar content and titratable acidity at distinct ripening stages in the ‘XM’. (A–D) respectively represent sucrose, fructose, glucose content and titratable acidity of ‘XM’ at three distinct ripening stages. The vertical bars represent the standard error of three biological replicates. Different letters in each column indicate significant difference between samples (*P* < 0.05).

As the fruit matures, ‘XM’ carambola changes its color from green to yellow (Fig. 6). We found that in Carambola fruits, sugar initially accumulates in the form of glucose and fructose, and later as sucrose, and observed a gradual increase in the levels of soluble sugars with fruits ripening. The soluble sugars in XM3 and XM2 were 71.33 mg g^−1^ FW and 45.27 mg g^−1^ FW, having 3.26 times and 2.07 times more soluble sugars compared with XM1, respectively (Fig. 7).

The content of organic acids during ‘XM’ fruits development followed an exact opposite trend to soluble sugars, i.e., as the fruit matured, the organic acid content gradually decreased. The organic acid content of XM3 fruit was 0.22%, only 20.75% of XM1 fruit (Fig. 7).

## Discussion

### Expression patterns of *AcSWEET2a/2b* and *AcSWEET16b* during fruit development

A desirable trait for Carambola fruit is a suitable ratio of soluble sugars to organic acids (Hu et al., 2005). We performed a systematic analysis of the expression of *AcSWEET* genes at three ripening stages: young fruits (XM1), developing fruits (XM2), and ripened fruits (XM3) at 40 d, 60 d, and 80 d post-flowering.

**Table 3:** Expression profiles of *AcSWEET* genes in Carambola fruits by RNA-Seq.

Gene name	Unigene ID	FPKM				
		Stage 1		Stage 2		Stage 3
		XM1		XM2		XM3
*AcSWEET1a*	c17276.graph_c0	2.97		1.87		2.27
*AcSWEET1b*	c17276.graph_c1	3.02		1.67		1.82
*AcSWEET2a*	c34256.graph_c0	6.14		4.68		2.79
*AcSWEET2b*	c35743.graph_c0	11.69		6.79		2.72
*AcSWEET16b*	c34763.graph_c0	21.51		28.45		28.16
*AcSWEET17*	c28577.graph_c0	1.00		1.62		1.14
*AcSWEET3*	c10581.graph_c0	0.00		0.13		0.19
*AcSWEET11*	c19732.graph_c0	0.16		0.07		0.14
*AcSWEET15*	c24551.graph_c0	0.09		0.33		0.10
*AcSWEET16a*	c104807.graph_c0	0.13		0		0

We found that the *AcSWEET16b* gene was highly expressed in all three stages of fruit development, and its expression was much higher compared with the other nine *AcSWEET* genes. Also, *AcSWEET16b* belonged to the clade IV category, which implied that it could transport both hexose and sucrose (Klemens et al., 2013; Xie et al., 2019), indicating that *AcSWEET16b* played an important fundamental role in Carambola fruit development.

The *AcSWEET2a/2b* genes belonged to the clade I category. In Arabidopsis, the *SWEET2* gene was mainly located in the vacuole membrane and transported hexose (Chen et al., 2015b). Interestingly, *AcSWEET2a/2b* showed the highest expression at the young fruit stage, which decreased as the fruit matured. This pattern was consistent with the expression pattern of *MdSWEET2.4* in apple (Wei et al., 2014) and *SlSWEET2a* in tomato (Feng et al., 2015). Also, it is known that in Carambola fruits, sugar initially accumulates in the form of glucose and fructose, and later as sucrose (Hu et al., 2005). Thus, it was inferred that *AcSWEET2a/2b* and *AcSWEET16b* synergistically participated in the transportation of sugar during the Carambola fruit development. Since sugar accumulation mainly occurred in the form of glucose and fructose during the initial and middle stages of fruit development, it exceeded the transport capacity of *AcSWEET16b*, which in turn, resulted in high expression of *AcSWEET2a/2b*. In the later stage of fruit development, sugar accumulation mainly occurred in the form of sucrose, which was easily accommodated by *AcSWEET16b*, leading to reduced demand for *AcSWEET2a/2b*, and thus their expression gradually decreased.

### *AcSWEET* gene family in Carambola

Recently, *SWEET* genes were analyzed in over 20 plant species, which indicated that they were involved in fruit development and ripening (Chen, 2014; Eom et al., 2015). However, there are no reports that analyzed the *SWEET* gene family in Carambola fruit. In this study, we identified and characterized *SWEET* genes in Carambola, comparing it with its homologous genes in Arabidopsis and grapevine, and transcriptome annotation information. Previous studies have reported that in higher plants, the number of reported *SWEET* genes varied from 7 to 108 and were found 17 in Arabidopsis (Chen et al., 2010), 17 in grapevine (Chong et al., 2014), 21 in rice (Yuan & Wang, 2013), 16 in litchi (Xie et al., 2019), 7 in loquat (Wu et al., 2017), and 108 in wheat (Gautam et al., 2019). In this study, we isolated 10 *AcSWEET* genes from the Carambola fruit, containing 229 aa to 300 aa, consistent with studies in other plants, such as 183-305 aa in loquat (Wu et al., 2017), 229-300 aa in litchi (Xie et al., 2019), 233-308 aa in tomato (Feng et al., 2015), 171-333 aa in banana (Miao et al., 2017), and 215-340 aa in apple (Zhen et al., 2018).

The phylogenetic analysis of the *SWEET* genes of Arabidopsis, Oriza, Vitis, and litchi, resulted in their division into four clades. However, based on our results, *AcSWEET* genes were classified into three clades: clade I, III, and IV, containing 5, 2, and 3 *AcSWEET* genes, respectively, but we did not find any *AcSWEET* genes in clade II. It could be attributed to the fact that the samples in this study were obtained only from fruits, not all organs of Carambola, and the *AcSWEET* genes were derived from the transcriptome database, not the genome.

### Transcriptome sequencing enriched Carambola sequence information

Ripe fruit of Carambola is known to be sweet, juicy, and slightly sour. The ratio of soluble sugar to organic acid has been shown to have a significant effect on the flavor of the carambola fruit (Hu et al., 2005). However, there is limited genomic data available for Carambola, which hinders the study of the molecular mechanisms of sugar and organic acid transport in Carambola fruits. Here, the fruit of ‘XM’, a sweet carambola variety, had high sugar content, good flavor, low acidity, and was analyzed by RNA-seq to explore the mechanism responsible for fruit flavor during fruit ripening.

Based on the sequencing results, 99,319 unigenes were generated, which were identical to the 119,701 unigenes of Chinese bayberry (Lin, Zhong & Zhang, 2019) and 82,036 unigenes of litchi (Lu et al., 2014). 51,642 (52.00%) unigenes sequences were annotated, which enriched the Carambola sequence database as well as provided basic data for subsequent research. However, as Carambola was a niche fruit, there was a lack of bioinformatics data on this fruit. As a result, 48.00% of unigenes sequences were not annotated, indicating that about half of the sequences had no apparent homologs, some of which were likely genes with novel functions. Thus, the Carambola unigenes library established in this study accumulated data that could be used for follow-up research on gene mining, cloning, and functional verification of important Carambola agronomic traits. It would also provide basic research data for the optimization and improvement of Carambola varieties and the economic value of Carambola.

## Conclusions

This work is the first report to establish the Carambola unigenes library, containing 99,319 unigenes with a minimum of 200 bp. Amongst these, a total of 51,642 unigenes (52.00%) were annotated. Additionally, we isolated and characterized ten *AcSWEET* genes and studies their structures, conserved motifs, and evolutionary relationships. The expression patterns of *AcSWEETs* during fruit ripening, combined with the soluble sugars content and the ratio of titratable acid, indicated that *AcSWEET2a/2b* and *AcSWEET16b* probably participated in sugar transport during fruit ripening. Thus, this study laid the foundation for further research on the functional role of the *AcSWEET* genes in the development of Carambola fruits.

##  Supplemental Information

10.7717/peerj.11404/supp-1 Supplemental Information 1 Primers used for RT-qPCR

10.7717/peerj.11404/supp-2 Supplemental Information 2 Length distribution of unigenes

10.7717/peerj.11404/supp-3 Supplemental Information 3 GO classification of Carambola unigenes

10.7717/peerj.11404/supp-4 Supplemental Information 4 The amino acid sequences used to generate phylogenetic tree

10.7717/peerj.11404/supp-5 Supplemental Information 5 The amino acid sequence similarities among the ten Carambola SWEETs

10.7717/peerj.11404/supp-6 Supplemental Information 6 Correlation (R2) of the expression levels of the 6 *AcSWEETs* genes measured by qRT-PCR and RNA-seq

10.7717/peerj.11404/supp-7 Supplemental Information 7 Raw data

## Additional Information and Declarations

### DNA Deposition

The following information was supplied regarding the deposition of DNA sequences: Transcriptome data are available at NCBI SRA: PRJNA647672, and this Transcriptome Shotgun Assembly project is available at DDBJ/EMBL/GenBank: GJAU00000000.1.

### Data Availability

The following information was supplied regarding data availability: Sequences are available at NCBI SRA: PRJNA647672. Additional raw data are available as a Supplemental File.
