# Supplementary material for: Identification and functional analysis of SWEET gene family in Averrhoa carambola L. fruits during ripening
Source: PeerJ. 2021 May 31;9:e11404. doi: 10.7717/peerj.11404 (PMC8174149; doi:10.7717/peerj.11404)
Supplement: Supplemental Information 1 [file peerj-09-11404-s001.docx]

Supplementary file 1

Primers used for qRT-PCR

| **Gene name** | **Forward primer** | **Reverse primer** | [**Product Size**](http://www.primer3plus.com/cgi-bin/dev/primer3plusHelp.cgi#PRIMER_PAIR_4_PRODUCT_SIZE) **(bp)** |
| --- | --- | --- | --- |
| AcSWEET1a | GGAGAGAGGGGAAGCATACATG | CTGTGGCCTTTGTCTCTCTCTT | 107 |
| AcSWEET1b | ATCTACCGCAACCACAAGGATT | AGGTAGTTCATTGCAGGACAGG | 184 |
| AcSWEET2a | CTCAGACCCACATTTCGGAGAA | CATACCACACGCAAATCAAGCA | 106 |
| AcSWEET2b | GCTTGGTCTGCGTTTCGAAG | ACAGTGGCTAACAGCATGCT | 200 |
| AcSWEET16b | AGAACAGAAGCCTCCGTAAAGG | ACAGTCGGCAGCAGTGTATTAA | 169 |
| AcSWEET17 | CATGATAATGTTGAGGCCTGCG | TCTTGGATGTAGGTGTCTTGGC | 113 |
| AcGAPDH | GTTGTCATTGAGGGCGATGC | AGCTGCCAAATACGACGACA | 166 |
