# Supplementary figures and images for: Identification and functional analysis of SWEET gene family in Averrhoa carambola L. fruits during ripening
Source: PeerJ. 2021 May 31;9:e11404. doi: 10.7717/peerj.11404 (PMC8174149; doi:10.7717/peerj.11404)

Supplementary file 2

Length Distribution of Carambola unigenes


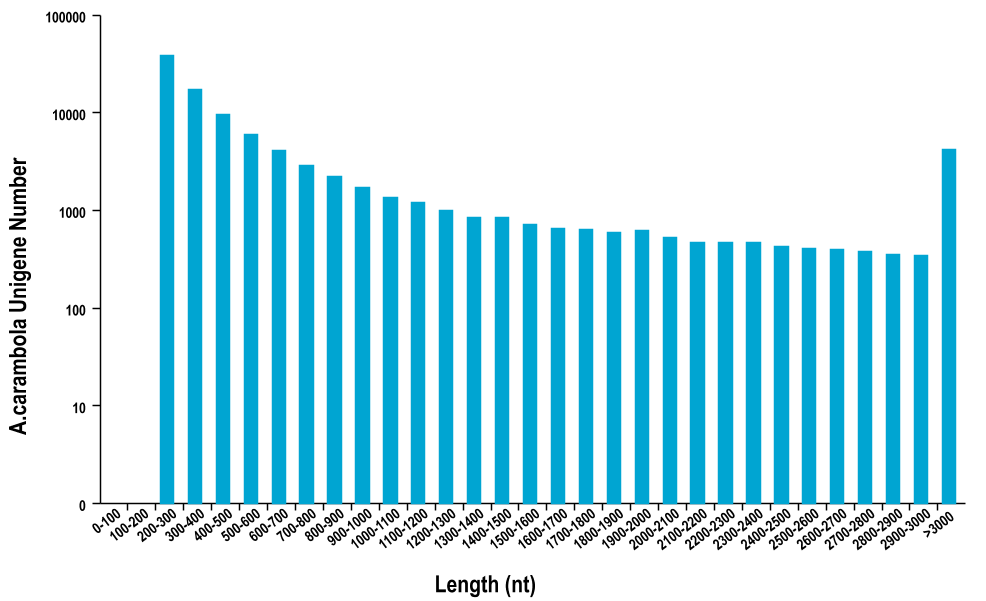

Supplement: Supplemental Information 2 [file peerj-09-11404-s002.docx]

Supplementary file 3

GO classification of Carambola unigenes


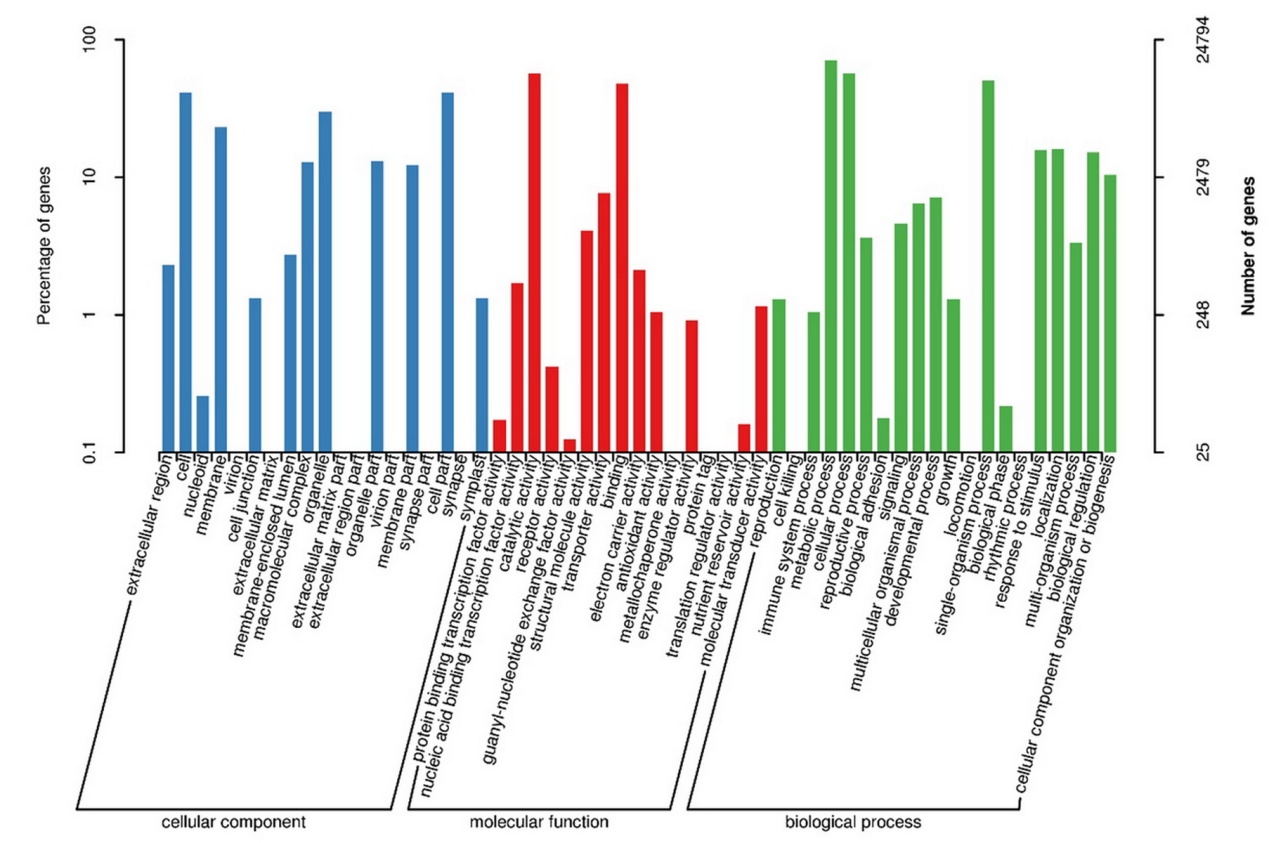

Supplement: Supplemental Information 3 [file peerj-09-11404-s003.docx]
