# Supplementary material for: Identification and functional analysis of SWEET gene family in Averrhoa carambola L. fruits during ripening
Source: PeerJ. 2021 May 31;9:e11404. doi: 10.7717/peerj.11404 (PMC8174149; doi:10.7717/peerj.11404)

Supplementary file 6

Correlation (R2) of the expression levels of the 6 *AcSWEETs* genes measured by qRT-PCR and RNA-seq


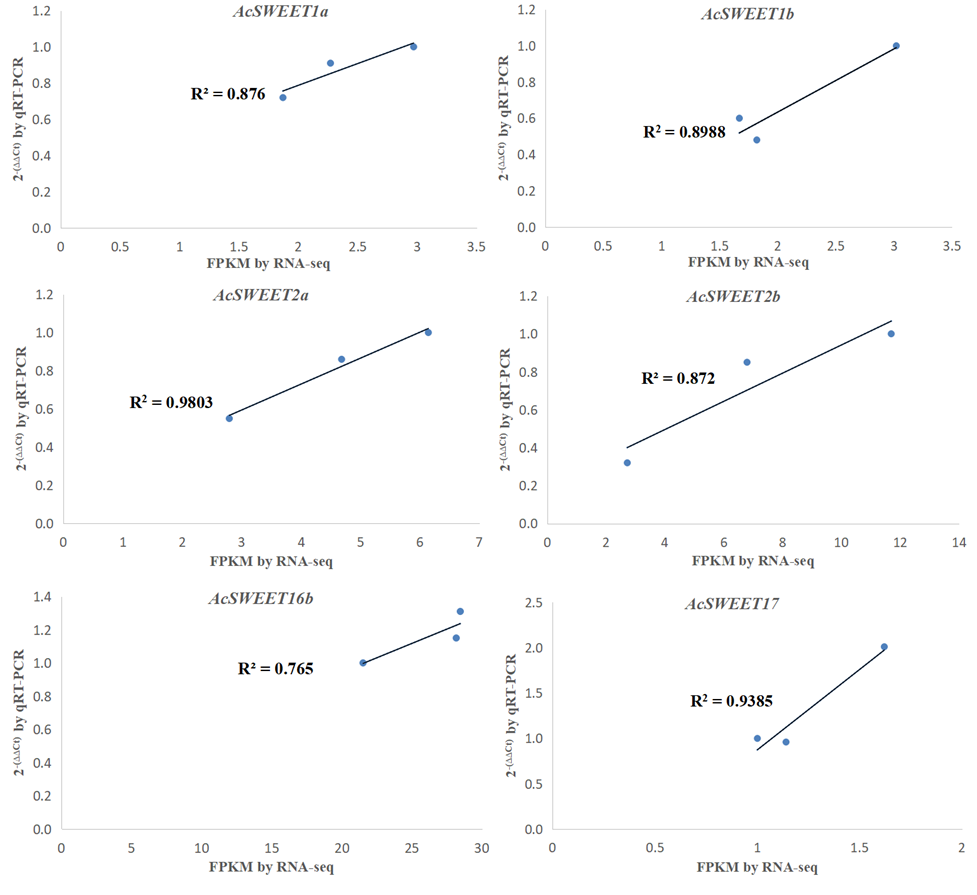

Supplement: Supplemental Information 6 [file peerj-09-11404-s006.docx]
